# Comparative Study of Biological Activities of Venom from Colubrid Snakes *Rhabdophis tigrinus* (Yamakagashi) and *Rhabdophis lateralis*

**DOI:** 10.3390/toxins9110373

**Published:** 2017-11-17

**Authors:** Yumiko Komori, Toru Hifumi, Akihiko Yamamoto, Atsushi Sakai, Manabu Ato, Kyoko Sawabe, Toshiaki Nikai

**Affiliations:** 1 Department of Microbiology, Faculty of Pharmacy, Meijo University, 150 Yagotoyama, Tenpaku-ku, Nagoya 468-8503, Japan; nikai@meijo-u.ac.jp; 2 Emergency Medical Center, Kagawa University Hospital, 1750-1 Ikenobe, Miki, Kita, Kagawa 761-0793, Japan; hifumitoru@gmail.com; 3 Department of Biosafety, National Institute of Infectious Disease, Gakuen 4-7-1, Musashimurayama, Tokyo 208-0011, Japan; yama-aki@niid.go.jp; 4 The Japan Snake Institute, Yabuzuka 3318, Ota, Gunma 379-2301, Japan; snake-b@sunfield.ne.jp; 5 Department of Immunology, National Institute of Infectious Disease, Toyama 1-23-1, Shinjuku, Tokyo 162-8640, Japan; ato@nih.go.jp; 6 Department of Medical Entomology, National Institute of Infectious Disease, Toyama 1-23-1, Shinjuku-ku, Tokyo 162-8640, Japan; sawabe@nih.go.jp

**Keywords:** *Rhabdophis tigrinus*, *Rhabdophis lateralis*, snake venom, prothrombin activator, blood coagulation

## Abstract

*Rhabdophis lateralis*, a colubrid snake distributed throughout the continent of Asia, has recently undergone taxonomic revisions. Previously, *Rhabdophis lateralis* was classified as a subspecies of *R. tigrinus* (Yamakagashi) until 2012, when several genetic differences were discovered which classified this snake as its own species. To elucidate the toxicity of venom from this poorly studied colubrid, various biological activities were compared between the venom from the two snake species. The components of their venom were compared by the elution profiles of reversed-phase HPLC and SDS-PAGE, and gel filtrated fractions were tested for effects on blood coagulation. Proteolytic activities of these fractions were also assayed by using synthetic substrates, fibrinogen, and matrix proteins. Similar to the *R. tigrinus* venom, the higher molecular weight fraction of *R. lateralis* venom contained a prothrombin activator. Both prothrombin time (PT) and activated partial thromboplastin time (APTT) of human plasma were shortened by the addition of *R. lateralis* and *R. tigrinus* venom. The thrombin formation was estimated by the uses of SDS-PAGE and chromogenic substrates. These venom fractions also possessed very specific proteinase activity on human fibrinogen, but the substrates for matrix metalloproteinase, such as collagen and laminin, were not hydrolyzed. However, there were some notable differences in reactivity to synthetic substrates for matrix metalloproteinase, and *R. tigrinus* venom possessed relatively higher activity. Our chemical investigation indicates that the components included in both venoms resemble each other closely. However, the ratio of components and proteolytic activity of some ingredients are slightly different, indicating differences between two closely-related snakes.

## 1. Introduction

*Rhabdophis lateralis*, a colubrid snake, has been considered to be subspecies of *R. tigrinus*, but has been recently reclassified as a different species by special reference to prominent geographical differentiation of the mitochondrial cytochrome *b* gene in Japanese populations [1]. *R. lateralis* is distributed over the Asian continent (primarily found in China) [2], whereas *R. tigrinus* (Yamakagashi) is widely distributed in Japan (exclusive of the northern island, Hokkaido). Both snakes are venomous snakes living in lowlands of rice paddies and watersides and aquatic animals. Especially in Japan, the number of individuals is decreasing, due to the influence of human development [3]. Because this snake has no grooved fangs, envenomation does not occur in most bites; therefore, this snake has long been considered non-venomous [3]. Although *R. tigrinus* bites induces life-threatening injuries, their mechanism and treatment have not been examined because of an extremely rare incidence of severe cases, compared with that of bites from *G. blomhoffii* and *P. flavoviridis*. Our former survey indicated that the pathophysiology of *R. tigrinus* bites was considered disseminated intravascular coagulation (DIC), with a fibrinolytic phenotype. However, the details of coagulation markers remain unknown. Moreover, although antivenom therapy prepared from hyper immunized horses (antivenin serum therapy) is established against *R. tigrinus* bites, sufficient information regarding antivenom therapy has not been provided in clinical practice [4,5]. Both snakes were listed in the WHO guidelines for the worldwide distribution of medically important venomous snake [6]. *R. tigrinus* antivenom was experimentally manufactured by horses, and is supported by Health Science Grants (1998–1999) from the Ministry of Health, Labour and Welfare in 2000 [3]. *R. tigrinus* venom is secreted from the Duvernoy’s gland, which is connected to the maxillary region immediately forward of the rearmost teeth. In the case of a rear-fanged snake such as *R. tigrinus*, it is difficult to collect much venom from a single snake, because there is no organ storing secreted venom, even if there is a venom gland. Furthermore, there is only one opportunity to collect venom from a single snake, because venom extraction must be carried out after euthanizing a snake, and separating the poison gland. On the other hand, in the case of front-fanged snake with poison bags, it is possible to collect venom without killing the snake by pressing the poison bag existing at the base of the fangs. In addition, multiple poisoning is possible from the front-fanged snake, by leaving the snake for a certain period of time. Due to this venom-secretory mechanism, it is very difficult to collect a large amount of venom to produce antivenom. We examined the possibility to use the venom of *R. lateralis*, that is widespread in China, for the antivenom production. For this purpose, a comparative analysis of the venoms of *R. lateralis* and *R. tigrinus* was carried out. In our analysis, a prothrombin activator, metalloproteinase, and cysteine-rich secretory protein (CRISP) were all identified as the venom components in both species, and some of their characteristics, such as venom toxicity, the proof of prothrombin activator and metalloproteinase, have been studied [6,7,8,9,10,11]. To elucidate the toxicity of *R. lateralis* venom, we tested various biological activities and compared their characteristics with *R. tigrinus* venom. Since colubrid venom is not well studied, as compared with the venom of front-fanged snakes such as *Sistrurus miliarius barbourin* and *Ophiophagus hannah*, our study contributes to the enhancement of knowledge of the venom proteomics of rear-fanged snakes [12,13].

## 2. Results and Discussion

### 2.1. Comparison of the Venom Components

**Figure 1:**
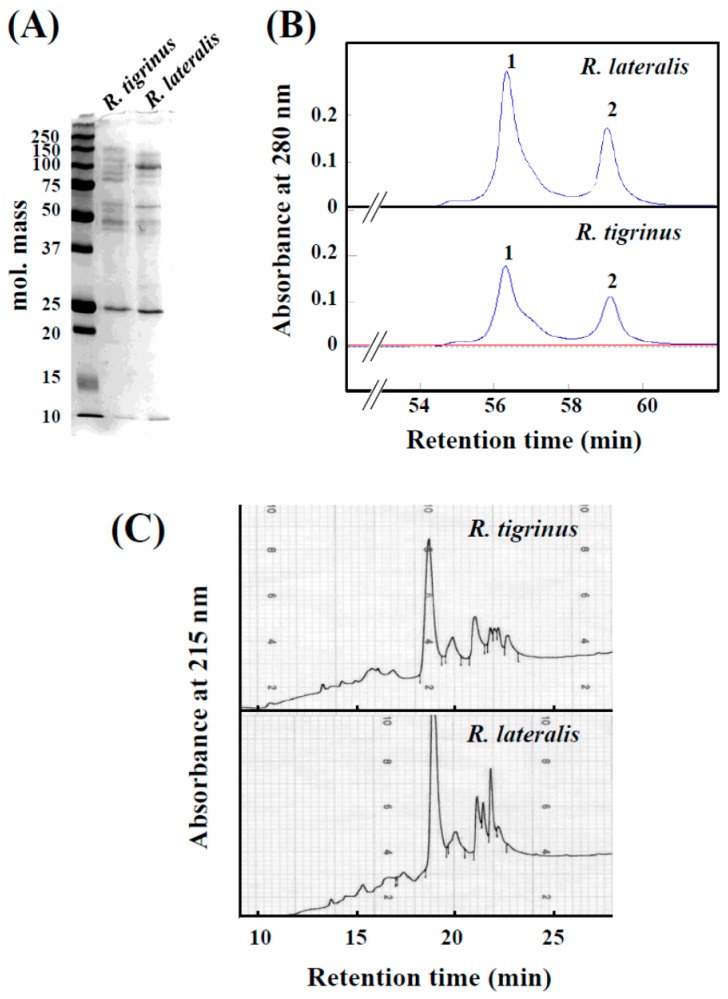
Comparison of crude venoms of *R. tigrinus* and *R. lateralis*. (**A**) SDS-PAGE of crude venoms; (**B**) Elution profiles from gel filtration column. Two milligrams of crude venoms were applied to the column and eluted with 0.01 M Tris-HCl buffer (pH 7.2) at a flow rate of 1.0 mL/min; (**C**) Elution profiles from a reversed-phase-HPLC column were obtained with a linear gradient from 20% to 90% acetonitrile over 25 min, with at a flow rate of 1.0 mL/min.

Crude venom of *R. tigrinus* and *R. lateralis* showed a similar electrophoresis pattern on SDS-PAGE, and in both cases, a band was observed with a molecular mass of 25 kDa (Figure 1A). On the other hand, in the molecular mass range of 37 to 150 kDa, similar bands were observed, but the densities of the bands appeared different.

When *R. lateralis* venom was applied to a gel filtration column, a similar elution pattern to *R. tigrinus* venom was obtained (Figure 1B). Several proteins which have the molecular mass of 50–150 kDa were observed on the SDS-PAGE of fraction 1 of both venoms (data not shown). Additionally, it was shown that protein of molecular mass 25 kDa was included in fraction 2. Coagulation activity for rabbit plasma was found in fraction 1 of both species.

The HPLC elution profiles indicate that the composition of both venoms is essentially the same, but found to be different in content (Figure 1C). The main peaks found in both venoms near 19 min of elution time were thought to be “tigrin” as determined from an *N*-terminal sequence analysis. It was also found that the band with molecular mass of 25 kDa is, indeed, tigrin from SDS-PAGE analysis (Figure 1A). Tigrin belongs to the CRISP (snake venom cysteine-rich secretory protein) family, which is widely distributed in various snake venoms, and that they inhibit smooth muscle contraction and cyclic nucleotide-gated ion channels [10,11].

### 2.2. Effect on the Blood Coagulation System

#### 2.2.1. Clotting Time of Human Plasma

*R. tigrinus* venom is well known to have strong clotting effects [7,8]. The existence of a prothrombin activator in the venom has been reported, and its activation mechanism was partially studied by Morita et al. [8]. To confirm whether *R. lateralis* venom provides a similar effect on the blood coagulation system, plasma clotting time was compared with *R. tigrinus* venom. As shown in Table 1, coagulation of human plasma was observed by the addition of venom (5 μg), and clotting time of 10 times diluted human plasma was measured to be 155 s and 168 s, respectively. The prothrombin time (PT), which evaluates the extrinsic pathway of coagulation, was also measured in the absence and presence of venom. The PT was shortened to 25 s from 33 s in the presence of venoms, and the difference was not found in the effects of both venoms. The activated partial thromboplastin time (APTT) in contrast to the PT, measures the activity of the intrinsic and common pathways of coagulation. The APTT was also decreased to 46–48 s from 109 s by the addition of venoms. Since each of PT and APTT was affected by both venoms, similar coagulation factors must be included in both *R. tigrinus* and *R. lateralis* venoms. Compared to other colubrid venom, *R. tigrinus* and *R. lateralis* venom possessed stronger clotting activity. Also, it is reported that some colubrid do not have prothrombin activator [14,15,16,17,18].

#### 2.2.2. Effect of Venom on Degradation of Prothrombin and Fibrinogen

**Figure 2:**
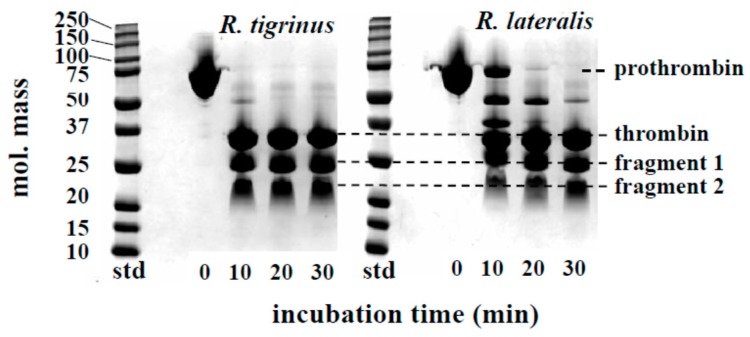
Time course of prothrombin degradation by *R. tigrinus* and *R. lateralis* venom. A mixture of human prothrombin (0.5 mg) and crude venom (5 μg) in a total volume of 110 μL 0.05 M Tris-HCl buffer, pH 7.5, containing 0.1 M NaCl was incubated at 37 °C. At the time intervals indicated, aliquots of the mixture were subjected to SDS-PAGE under unreduced conditions.

The time course of prothrombin degradation is shown in Figure 2. Both venoms hydrolyzed prothrombin, and the band of approximately 35 kDa appeared, suggesting that thrombin was formed within 10 min. At the same time, the bands which estimated to be *N*-terminal fragment 1 and fragment 2 of prothrombin were also detected. The activation process of prothrombin by *R. tigrinus* venom had been reported by Morita et al. [8]. The degradation of prothrombin caused by *R. lateralis* venom was delayed approximately 10 min compared to *R. tigrinus* venom, but the final products were almost equivalent. These results indicate that the effects of prothrombin activators in both venoms are considered to be the same. Regarding the degradation of prothrombin and fibrinogen, there are several reports that colubrid snake containing *R. tigrinus* and *R. lateralis*, to a greater or lesser extent, has these activities [14,15,16,17,18].

For further confirmation that thrombin was formed, the hydrolytic activity was examined by using chromogenic substrates S-2238 for thrombin and S-2222 for factor Xa. Briefly, these substrates were added to the mixture of plasma and venom fraction 1 (Figure 1B), as described in Section 4. The mixture of normal plasma and fraction 1 of both venoms remarkably hydrolyzed S-2238, but most of S-2222 was not hydrolyzed (Table 2). These data indicate that thrombin had been formed in the mixture, and factor Xa was not formed. When factor II (prothrombin)-deficient plasma was used for the assay, the hydrolysis amount of S-2238 was clearly decreased from 1001.7 to 170.0 (*R. tigrinus*) and from 1493.4 to 214.0 (*R. lateralis*), respectively. As to the reason of some hydrolysis activity of S-2238 under these conditions, a trace of factor II (prothrombin) (<3% of normal plasma) might exist in plasma. When the venom fraction 2 was used for the assay, these substrates were not hydrolyzed. Regarding other colubrids, there is no report that measured hydrolysis activity using the substrates which we used in this study [14,15,16,17,18].

**Figure 3:**
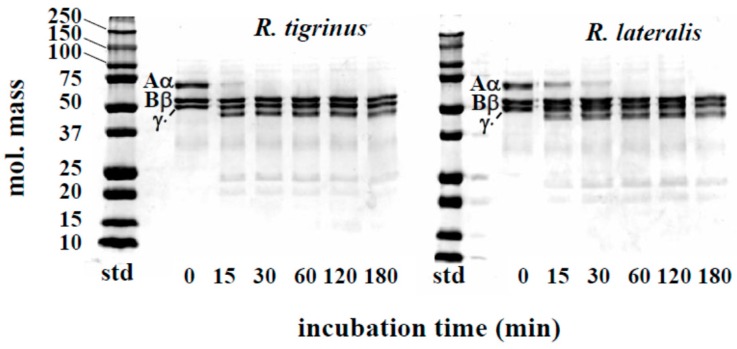
Time course of fibrinogen degradation by *R. tigrinus* and *R. lateralis* venom. A mixture of human fibrinogen (0.1 mg) and venom fraction 1 (5 μg) in a total volume of 150 μL 0.01 M Tris-HCl buffer, pH 7.5, containing 0.1 M NaCl, was incubated at 37 °C. At the time intervals indicated, aliquots of the mixture were subjected to SDS-PAGE under reduced conditions.

The effect of venom fraction 1 on the blood coagulation system was also measured by using human fibrinogen. Although the Aα chains of fibrinogen were rapidly hydrolyzed by *R. tigrinus* and *R. lateralis* venoms, Bβ and γ chains were not degraded (Figure 3). As to the time course of fibrinogen degradation by colubrid venom, *Philodryas patagoniensis* possessed stronger activity than both *R. tigrinus* and *R. lateralis* [16]. It is not clear now what these fibrinogenolytic effects are due to, but a proteolytic activity described in a later part of this paper (Section 2.3) may be associated with these phenomena.

From the mentioned results, the strong blood clotting effect of *R. lateralis* venom is thought to be due to prothrombin activation in the same way as *R. tigrinus* venom. In addition, the fibrinogenolytic activity found in both venoms might aggravate the disturbance of the blood coagulation system more.

### 2.3. Proteolytic Activity

#### 2.3.1. Effects of Venom Fractions on Synthetic Substrates

Fluorescence quenching substrates designed for matrix metalloproteinase (MMP) were employed to determine the proteolytic activities of venom fractions (Table 3). NFF-2 and NFF-3 are the substrates for stromelysin-1 (MMP3), and 3163v is for matrilysin (MMP7). NFF-3 and 3163v were hydrolyzed by fraction 1 of both venoms. Fraction 1 from *R. lateralis* venom also possessed hydrolytic activity on NFF-2, however, the effect of *R. tigrinus* venom on this substrate was relatively low. The proteolytic activity for these substrates was not found in fraction 2 of either venom.

#### 2.3.2. Effects of Venom Fractions on Extracellular Matrix Proteins

Collagen type IV and laminin are the most common proteins found in the extracellular matrix and constitute the basement membrane. Therefore, the degradation of collagen by snake venom is important in the generation of necrosis. Since fraction 1 hydrolyzed NFF-3, the proteolytic activity for collagen type IV and laminin (substrates of MMP3) were also examined. However, degradation of these matrix proteins was not observed (data not shown). These results indicate that the proteolytic effects of venom components are substrate-specific, and only prothrombin and fibrinogen are hydrolyzed efficiently. Since the small molecular weight peptides were easily affected by proteases, hydrolysis of synthetic substrates may have been observed (Table 3). Compared to *R. tigrinus* and *R. lateralis*, other colubrids had variety of proteolytic activities such as caseinase, kallikrein, collagenase, and exopeptidase, etc. [15,16].

#### 2.3.3. Comparison of Proteolytic Specificity

Since the comparison of the cleavage sites of the oxidized insulin B chain had been used to examine specificity of various proteinases, this substrate was employed to determine the cleavage specificity of both venoms. The digested peptide fragments formed by the fraction 1 of both venoms were separated with reversed-phase HPLC and analyzed by MALDI-TOF MS. The amino-terminal sequences of digested fragments were also determined (Table 4).

When the fraction 1 of *R. tigrinus* venom was incubated with the insulin B chain, four fragments were obtained by reversed-phase HPLC. Two cleavage sites were determined from the analyzed data and they were Ala(14)–Leu(15) and Tyr(16)–Leu(17). Fraction 1 of *R. lateralis* venom also cleaved the same sites, however, additional cleavage sites of His(10)–Leu(11), Leu(17)–Val(18), and Val(18)–Cys(19) were observed. These results clearly show that there is some difference between *R. tigrinus* and *R. lateralis* venoms. Since *R. lateralis* venom hydrolyzed more MMP substrates nonspecifically (Table 3), and degraded the insulin B chain in more sites, this venom may possess a stronger influence for tissues by actual envenomation.

## 3. Conclusions

It is shown that the composition of *R. lateralis* venom is similar to that of *R. tigrinus*; however, some differences were clearly shown in our investigation. The presence of prothrombin activator, proteinase with fibrinogenolytic activity and CRISP were found in both venoms. There are few reports about the clinical damage caused by an envenomation of *R. lateralis*. However, the venom components described in this study had a profound effect on the blood coagulation system, thus, a serious pathologic effect may occur if its venom is injected into a blood vessel. It is fortunate that there is a common antigen, although the two venoms have some differences, such as *Protobothrops flavoviridis* and *Protobothrops elegans* [19]. This is important from the viewpoint of colubrid snakebite treatment. Based on the results of this study, it was suggested that the possibility of using *R. lateralis* venom was opened as an immunogen of *R. tigrinus* antivenom, but there are many items to be confirmed in the future.

## 4. Materials and Methods

### 4.1. Materials

Crude *R. tigrinus* venom was obtained from the Japan Snake Institute, because *R. tigrinus* is the only species from that genus *Rhabdophis* in Japan. On the other hand, several kinds of *Rhabdophis* inhabit China, but only *R. lateralis* is found within the Shanghai circumference. *R. lateralis* were collected by Dr. Jiuru Sun of the Shanghai Serum Bio-tech Co., Ltd. (Shanghai, China) within two weeks. *R. lateralis* venom was extracted immediately after collecting snakes at the company’s laboratory. Prior to the collection of venom, the snakes were given only water without feeding to avoid any potential effect of feeding on venom composition. To obtain crude venom, we had done following three steps: (1) Collect the relatively large 16 *R. lateralis* wild snakes and 20 *R. tigrinus* wild snakes with a body length of around 1 m and identify each snake sample. At that time, we only identify the type of snake, we have not confirmed sex, age etc.; (2) Extract Duvernoy’s gland from euthanized snakes and rapidly freeze it; (3) Extract with physiological saline, lyophilize to extract the extracted venoms for each type of snake, and put together for long term storage.

Standard human plasma was purchased from Siemens Healthcare Diagnostics Products GmbH (Marburg, Germany) and congenital factor II-deficient plasma was from George King Bio-Medical, Inc. (Overland Park, KS, USA). Rabbit plasma for plasma coagulant assay was the product of Eiken Chemical Co., Ltd. (Tokyo, Japan). In vitro diagnostic reagents for determination of prothrombin time; Coagpia^®^ PT-N, and for activated partial thromboplastin time; Coagpia^®^ APTT-N were the products from Sekisui Medical Co., Ltd. (Tokyo, Japan). Human fibrinogen and oxidized insulin B chain were supplied by Sigma-Aldrich Co., Ltd. (Dorset, UK), and human prothrombin was from Enzyme Research Laboratories Inc. (South Bend, IN, USA). Laminin was obtained from Wako Pure Chemical Industries Ltd. (Osaka, Japan), and collagen type IV was from Nitta Gelatin Inc. (Osaka, Japan). Chromogenic substrate for factor Xa; S-2222 (Bz–Ile–Glu–Gly–Arg–*p*NA) and substrate for thrombin; S-2238 (H–*D*-Phe–Pip–Arg–*p*NA) were the products of Chromogenix (West Chester, OH, USA). The fluorescence-quenching substrate for matrix metalloproteinases; NFF-2 (MCA–Arg–Pro–Lys–Pro–Tyr–Ala–Nva–Trp–Met–Lys(Dnp)–NH_2_), NFF-3 (MCA–Arg–Pro–Lys–Pro–Val–Glu–Nva–Trp–Arg–Lys(Dnp)–NH_2_), 3163v (MCA–Pro–Leu–Gly–Leu–Dpa–Ala–Arg–NH_2_), and the reference compound MOCAc–Pro–Leu–Gly were purchased from Peptide Institute, Inc. (Osaka, Japan). α-Cyano-4-hydroxycinnamic acid (HCCA), a matrix substance for MALDI-TOF MS and peptide calibration standard were obtained from Bruker Daltonics (Yokohama, Japan). Other chemicals used were of analytical grade and purchased from commercial sources. A gel filtration column, Enrich SEC 70, for BioLogic DuoFlow system was obtained from Bio-Rad Laboratories, Inc. (Hercules, CA, USA), and Develosil^TM^ ODS-HG-5 (4.6 × 250 mm) for reversed-phase HPLC was from Nomura Chemical Co., Ltd. (Seto, Japan).

### 4.2. Fractionation of Crude Venom

Crude venoms of *R. tigrinus* and *R. lateralis* (2 mg each) were applied to an Enrich SEC 70 gel filtration column (10 × 300 mm) which was connected to a BioLogic DuoFlow system, and eluted with 0.01 M Tris-HCl buffer (pH 7.2) at a flow rate of 1.0 mL/min. Crude venoms were also fractionated by a reversed-phase HPLC column (ODS-HG-5) equilibrated with 0.1% trifluoroacetic acid in 80% H_2_O and 20% acetonitrile. Elution was achieved over 25 min with a linear gradient from 20% to 90% acetonitrile, at a flow rate of 1.0 mL/min.

### 4.3. Blood Coagulation Assays

According to the instruction manual of the supplier, prothrombin time (PT) and activated partial thromboplastin time (APTT) were measured. To measure excessive coagulation exactly, diluted human plasma was used for these assays. Fifty microliters of human plasma and appropriate concentration of venom fractions in 0.45 mL of saline were mixed and kept at 37 °C. PT was measured by adding 50 μL of thromboplastin reagent into the mixture described above. APTT was assayed by adding 50 μL of APTT reagent and 50 μL CaCl_2_ solutions subsequently into the plasma–venom mixture. The assay process was conducted according to the instructions of the manufacturer and performed at 37 °C.

The hydrolytic activity for blood coagulation factors of venom fraction was measured by using chromogenic substrates [20,21]. Briefly, 50 μL of venom solution was added to the mixture of plasma (50 μL), 50 mM Tris-HCl buffer (pH 8.3) containing 227 mM NaCl (360 μL), and 0.5 M CaCl_2_ (50 μL). After the incubation for 1 min at 37 °C, 0.5 mL of 1.59 mM substrate solution was added and the rate of pNA formation was determined by measuring the increase in absorbance per min at 405 nm.

### 4.4. Proteolytic Activities

The hydrolytic activity for matrix metalloproteinase was assayed by using synthetic fluorescence quenching substrates [22,23]. Substrates were prepared as 10 mM stock solutions in dimethyl sulfoxide. Assay was carried out by incubating 5 μL of substrate stock solution with 10 μL of venom fraction in 50 mM Tris-HCl buffer (pH 7.5) containing 0.15 M NaCl, 10 mM CaCl_2_, 0.05% Brij 35, and 0.02% NaN_3_. The reaction was stopped by the addition of 0.9 mL of 3% acetic acid, and the intensity of fluorescence was measured at *λ*_ex_ of 325 nm and *λ*_em_ of 393 nm. The amount of substrate hydrolyzed was calculated from the standard curve of the reference compound, MOCAc–Pro–Leu–Gly.

The effect of venom on human prothrombin, fibrinogen, collagen, and laminin were measured by monitoring the time course degradation of these proteins on SDS polyacrylamide gel. Oxidized insulin B chain was used for the determination of enzyme specificity. Briefly, the hydrolyzed peptides were fractionated by a reversed-phase HPLC column (Develosil 300 ODS-7), and the fragments were identified by MALDI-TOF MS spectrum using Autoflex speed (Bruker Daltonics). The *N*-terminal amino acid sequence of each fragment was also detected by a Procise protein sequencing system (Applied Biosystems, Foster City, CA, USA).

## Figures and Tables

**Table 1:** Coagulation assay of human plasma.

Assay Condition	Clotting Time (s) *	
**Control (10× normal plasma)**		
Prothrombin time ^†^	33 ± 1.2	
Activated partial thromboplastin time ^‡^	109 ± 0.8	
**Plasma (10×) + 5 μg of Venom**	***R. tigrinus***	***R. lateralis***
Clotting time without reagents ^§^	155 ± 3.7	168 ± 11.1
Prothrombin time ^†^	25 ± 0.5	25 ± 0.5
Activated partial thromboplastin time ^‡^	48 ± 4.5	46 ± 3.3

* The data shown represent the average of three experiments ± SD. ^†^ Prothrombin time was measured by adding 50 μL of thromboplastin reagent into the 10 times diluted human plasma in the absence and presence of venom. ^‡^ Activated partial thromboplastin time was assayed by adding 50 μL of APTT reagent and 50 μL CaCl_2_ solution subsequently into the 10 times diluted plasma in the absence and presence of venom. ^§^ Clotting time was determined simply by adding venom.

**Table 2:** Thrombin and factor Xa formation assay from human plasma.

Substrate	Activity (ΔA_405 nm_/min/mg Protein)	
	*R. tigrinus*	*R. lateralis*
	Fraction 1	Fraction 1
**normal plasma**		
S-2222 *	3.2	4.2
S-2238 ^†^	1001.7	1493.4
**factor II-deficient plasma** ^‡^		
S-2238	170.0	214.0

* Bz–Ile–Glu–Gly–Arg–*p*NA. ^†^ H–*D*-Phe–Pip–Arg–*p*NA. ^‡^ Factor II activity was determined as <3%.

**Table 3:** Proteolytic activity of venom fractions on fluorescence-quenching substrate for matrix metalloproteinases.

Substrate	Proteolytic Activity (nmole Substrate Hydrolyzed/min/mg Protein)			
	*R. tigrinus*		*R. lateralis*	
	Fraction 1	Fraction 2	Fraction 1	Fraction 2
NFF-2 *	0.043	0	0.327	0
NFF-3 ^†^	0.441	0	0.283	0
3163v ^‡^	0.431	0.010	1.287	0

* MCA (7-methoxycoumarin-4-yl)acetyl)–Arg–Pro–Lys–Pro–Tyr–Ala–Nva–Trp–Met–Lys(Dnp)–NH_2_. ^†^ MCA–Arg–Pro–Lys–Pro–Val–Glu–Nva–Trp–Arg–Lys(Dnp)–NH_2_. ^‡^ MCA–Pro–Leu–Gly–Leu–Dpa–Ala–Arg–NH_2_.

**Table 4:** Molecular mass and *N*-terminal amino acid sequence of digestion fragments of oxidized insulin B chain *.

Digested Fragment	By *R. tigrinus* Fraction 1		By *R. lateralis* Fraction 1	
	*m*/*z*^†^	*N*-terminal Sequence ^‡^	*m*/*z*^†^	*N*-terminal Sequence ^‡^
fragment 1	n.d. ^#^	–	1189.5	F V N
fragment 2	1601.9	F V N	1601.8	F V N
fragment 3	n.d. ^#^	–	1522.7	V C G
			1423.7	C G E
fragment 4	1635.8	L V C	1635.7	L V C
fragment 5	1878.1	F V N	1877.9	F V N
fragment 6	1912.0	L Y L	1911.9	L Y L

* Oxidized insulin B chain; Phe–Val–Asn–Gln–His–Leu–Cys(SO_3_H)–Gly–Ser–His–Leu–Val–Glu–Ala–Leu–Tyr–Leu–Val–Cys(SO_3_H)–Gly–Glu–Arg–Gly–Phe–Phe–Tyr–Thr–Pro–Lys–Ala. ^†^ Mass-to-charge ratio obtained from MALDI-TOF MS spectral analysis. ^‡^ The amino-terminal residues were analyzed by a Procise protein sequencing system. ^#^ n.d.; fragment was not detected.
